# Bullying as a Social Risk Factor for Neurobiological Vulnerability to Adolescent Anxiety: An Exploratory Study

**DOI:** 10.1192/j.eurpsy.2026.10252

**Published:** 2026-07-31

**Authors:** D. Porta-Casteràs, R. Miranda-Olivos, V. De la Peña-Arteaga, O. Contreras-Rodríguez, A. Álvarez-Monell, M. Guxens, M. Vrijheid, N. Cardoner, M. Cano

**Affiliations:** 1 Sant Pau Mental Health Research Group, Institut de Recerca Sant Pau (IR SANT PAU), Barcelona; 2 Department of Psychiatry and Forensic Medicine, Universitat Autònoma de Barcelona (UAB), Bellaterra; 3 ISGlobal, Barcelona, Spain

## Abstract

**Introduction:**

Bullying is one of the most common adverse social experiences during early adolescence. Identifying potential brain-based biomarkers that modulate vulnerability to internalizing symptoms after bullying could inform targeted prevention strategies.

**Objectives:**

To identify potential neuroimaging biomarkers associated with subclinical anxiety, depressive, and obsessive-compulsive symptoms in adolescents who were exposed to bullying.

**Methods:**

We collected neuroimaging and subclinical mental health data at the 18-year follow-up of the INMA-Sabadell cohort. Among 159 healthy adolescents without neuropsychiatric or neurodevelopmental disorders, a subgroup of 12 adolescents reported bullying exposure at age 11 (Olweus Bully/Victim Questionnaire). An age- and sex-matched control subsample of 12 non-victims was selected. Anatomical, functional, and diffusion magnetic resonance imaging were processed with FreeSurfer, MELODIC, and TRACULA to derive cortical surface area and thickness, subcortical volumes, functional connectivity, and diffusion metrics. Subclinical symptoms were assessed with the Obsessive-Compulsive Inventory-Child Version and the Depression, Anxiety, and Stress Scale (DASS). Symptom–brain associations were compared between bullying victims and non-victims using Fisher Z-transformed Pearson correlations (structural data) or multivariate ANOVA (functional data).

**Results:**

Adolescents exposed to bullying at age 11 showed a stronger positive relationship between left inferior parietal lobule (IPL) surface area and DASS-Anxiety scores at age 18 compared to non-victims (Z=5.01; *p_Bonf_*=0.0004). Within-group analyses found a significant positive correlation in victims (R(9)=0.94; *p_Bonf_*=0.00086), whereas non-victims exhibited a non-significant negative correlation (R(9)=-0.53; *p_Bonf_*=1).

**Image 1:**
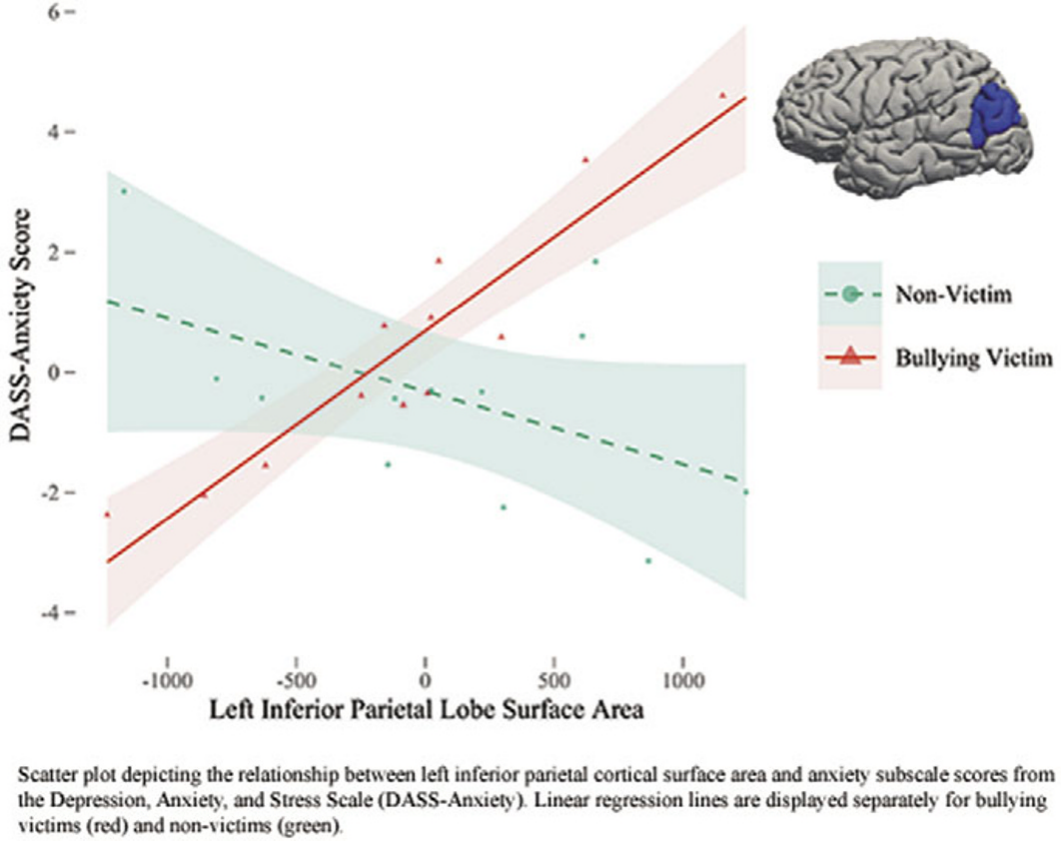
Image 1: Long description.

**Conclusions:**

Preadolescent bullying may modulate IPL-anxiety coupling, suggesting that early social adversity can leave lasting imprints on brain–anxiety relationships. The IPL, implicated in social cognition, blame attribution, and attentional processing of unexpected stimuli, has also been related to polygenic risk for neuroticism, with previous research pointing to an unidentified environmental moderator. Our exploratory study highlights bullying as a potential environmental risk factor shaping neurobiological vulnerability to adolescent internalizing symptoms.

**Disclosure of Interest:**

None Declared

## Long descriptions

### Image 1: Long description

A scatter plot with linear regression lines and shaded confidence intervals.

* Axes: The x-axis represents Left Inferior Parietal Lobe Surface Area ranging from minus 1000 to 1000. The y-axis represents D A S S-Anxiety Score ranging from minus 4 to 6.

* Anatomical Inset: In the top-right corner, a 3D model of a human brain is shown from a lateral view with the left inferior parietal lobe highlighted in blue.

* Legend: Located on the right, green circles with a dashed line represent Non-Victim. Red triangles with a solid line represent Bullying Victim.

* Data Trends:

- The Bullying Victim group shows a strong positive linear correlation. The solid red line starts at approximately x equals minus 1200, y equals minus 3 and rises steeply to x equals 1200, y equals 4.5.

- The Non-Victim group shows a slight negative linear correlation. The dashed green line starts at approximately x equals minus 1200, y equals 1 and descends gradually to x equals 1200, y equals minus 2.

- The two regression lines intersect near the center of the plot where x is approximately minus 250 and y is 0.

Navigate back to Image 1:.

